# Ubiquitination-dependent, proteasomal degradation of the retroviral oncoprotein Tax by niclosamide, an anti-helminthic molecule

**DOI:** 10.1186/1742-4690-11-S1-P148

**Published:** 2014-01-07

**Authors:** Di Xiang, Yunsheng Yuan, Li Chen, Chandra Belani, Hua Cheng

**Affiliations:** 1 Institute of Human Virology, University of Maryland School of Medicine, Baltimore, MD, USA; 2 Penn State Hershey Cancer Institute, Hershey, PA, USA

## 

Adult T cell leukemia and lymphoma (ATL) is a highly aggressive form of hematological malignancies, which is caused by chronic infection of human T cell leukemia virus type 1 (HTLV-1). The viral genome encodes an oncogenic protein Tax that plays a key role in trans activating viral gene transcription and in deregulating cellular oncogenic signaling to promote survival and proliferation of virally infected T cells. Hence, Tax is a desirable therapeutic target, particularly at early stage of HTLV-1-mediated oncogenesis. We here show that niclosamide, an anti-helminthic molecule, induced apoptosis of HTLV-1-transformed T cells. Niclosamide promoted formation of polyubiquitinated Tax protein aggregate, facilitating its subsequent degradation in proteasome. Consistent with niclosamide-mediated Tax degradation, the transcription of HTLV-1 viral genes is suppressed. Furthermore, niclosamide inhibited the activities of MAPK/ERK and Stat3, and down regulated pro-survival Bcl-2 family members such as Mcl-1. Since Tax, Stat3 and Mcl-1 are growth-promoting molecules in HTLV-1-transformed T cells, our data demonstrated a novel mechanism of niclosamide in inducing polyubiquitination-dependent degradation of Tax and certain cellular pro-survival molecules, thereby promoting apoptosis of HTLV-1-associated leukemia.

